# Challenges in colonic stenting: Giving up is not an option

**DOI:** 10.1111/den.14436

**Published:** 2022-10-03

**Authors:** Toshio Kuwai, Takeshi Mizumoto, Yuzuru Tamaru, Ryusaku Kusunoki, Sauid Ishaq

**Affiliations:** ^1^ Department of Gastroenterology, National Hospital Organization Kure Medical Center and Chugoku Cancer Center Hiroshima Japan; ^2^ Gastroenterology Department, Dudley Group Hospitals Birmingham City University Birmingham UK; ^3^ St George's University Grenada, West Indies

The placement of a self‐expandable metallic stent (SEMS) is considered effective for decompressing malignant colonic obstruction, both as a palliative treatment modality and as a bridge to surgery (BTS).^1^, ^2^ Through‐the‐scope (TTS) placement of SEMS has become the standard method for colonic stenting, achieving a high technical success rate.^3^ However, technical challenges can occur in patients with intrapelvic adhesions caused by nonmalignant peritonitis or a surgery, peritoneal carcinomatosis, a score of 0 on the ColoRectal Obstruction Scoring System,^4^ expansive strictures, or a tumor site in the right colon.^3^ Additionally, the European Society of Gastrointestinal Endoscopy (ESGE) guidelines suggest that performing colorectal stenting is relatively difficult in patients with peritoneal metastases or tumors close to the anal verge (<5 cm).^2^ However, recently developed innovations in devices and procedures can overcome most of these problems. Therefore, all endoscopists should be aware of and implement these innovations into their practice to improve outcomes and reduce adverse events.

Self‐expandable metallic stent placement in malignant colonic obstruction with peritoneal carcinomatosis is particularly challenging given the associated narrowing, adhesion, and tortuosity of the involved colon. The increased mobility of the bowel in such instances restricts endoscopic insertion and operation. Moreover, it may be difficult to access or accurately identify sites of stenosis secondary to tumor invasion from the serosa.^3^ Consequently, colonic stenting for tumors with peritoneal carcinomatosis is associated with decreased rates of clinical success and increased rates of adverse events.^2^ However, the lower rates of morbidity and mortality achieved using endoscopic stenting in such patients would make stenting preferable to surgery. In this issue of *Digestive Endoscopy*, Iboshi *et al*.^5^ presented a novel salvage procedure of colonic stenting in demanding circumstances such as concomitant peritoneal carcinomatous. This salvage procedure is described as “over‐the‐catheter endoscope replacement” (OCER). The authors have shown it to be particularly effective for cases in which peritoneal carcinomatosis prevents access to the site of the obstruction by the large‐caliber colonoscope, which is normally used for SEMS placement in the TTS method. In this OCER technique, an ultra‐slim endoscope is inserted through a tortuous segment of colon to advance a guidewire beyond the obstruction site. Then the slim endoscope is replaced by a large‐caliber colonoscope that is inserted over the guidewire, and colonic stenting is performed using the TTS method. This cutting‐edge OCER technique will be suitable for overcoming challenging circumstances in colonic stenting.

There is no doubt that basic procedures are essential for complex cases in the colonic stenting field. The Japan Colonic Stent Safe Procedure Research Group, an affiliate of the Japan Gastroenterological Endoscopy Society, published safety instructions for colonic stenting in 2012.^4^ The significant points of the procedure are as follows:

1. The procedure is conducted in the fluoroscopy room, and during insertion of a SEMS through a colonoscope, positioning is performed under radiographic guidance.
2. The procedure is performed only after an excellent visual field has been established; placement is impossible when the visual field is obscured, similar to the case with hemorrhage.
3. Before SEMS placement, the endoscopic retrograde cholangiopancreatography (ERCP) technique is used to advance a guidewire through a sheath.
4. Neither balloon nor bougie dilatation of the stenotic region is performed.
5. The distal end of the stenotic lesion is marked with metal clips.
6. Prophylactic SEMS placement should not be performed.

This advice led to a technical success rate of approximately 98% for colonic stenting,^3^ implying that colonic stenting could be successfully performed in most circumstances. Additionally, it has been claimed that the perforation rate in BTS cases can be reduced to <2%, thus improving safety.^1^ However, despite adherence to standards and basic procedures, successful colonic stenting remains challenging in some cases.^3^

Colonic stenting was initially reported in Japan in 1991 by Dohmoto,^6^ and since then, stent technology has advanced. Currently, most stents are composed of a shape‐memory alloy of nitinol. The structure of the SEMS has also been modified: in particular, a mesh‐weave SEMS maintains adequate radial force with weak axial force, lessening the possibility of perforation.^7^, ^8^ A re‐retractable function has been developed that facilitates the positional adjustment of SEMS. The availability of SEMS on a 9F delivery system while maintaining important SEMS properties allows placement with a highly flexible‐tipped small caliber colonoscope with a working channel of 3.2 mm,^8^ facilitating colonic stenting in challenging circumstances such as at sharp angles.

The placement of colonic SEMS for tumors close to the anal verge is recognized as challenging because of undesirable consequences and tenesmus associated with closeness to the dentate line. To prevent these adverse events, deploying SEMS precisely at the distal edge of a tumor close to the anal edge is crucial. Because of these technical challenges, the ESGE guidelines consider the presence of tumors close to the anal verge (<5 cm) as a relative contraindication for stenting. Most of these patients are treated with stoma creation instead. The newly developed proximal release‐type colonic stent can be used in such cases.^9^ This stent is placed using the over‐the‐wire technique while the proper stance is maintained under endoscopic view to keep the distal edge of the stent strictly at the distal edge of the tumor. Using this procedure, a proximal release‐type colonic stent can be successfully placed for tumors located at a distance of <2 cm from the anal verge without any adverse events.^9^

Various ancillary techniques have also been developed to overcome several challenges in colonic stenting. Use of CO_2_ insufflation, changing the patient's position, applying abdominal pressure, and using deep breathing are among the fundamental principles for achieving increased success. In addition to the OCER technique, the sequential use of double‐balloon endoscopy (DBE) and ultra‐slim endoscopy has been reported in difficult stenting.^10^ In this technique, first, the DBE is placed into an obstruction site and then the endoscope is withdrawn, leaving only the over tube with an inflated balloon. Subsequently, an ultra‐slim endoscope is inserted through the over tube, passing through the obstruction site to enable the placement of a guidewire. This technique works well for the placement of guidewires in challenging circumstances, such as malignant right colonic obstruction.

In this editorial, we described the advances in novel devices and techniques for difficult colonic stent placement, which can help endoscopists overcome the challenges in complex cases. Further development of safer and more dependable procedures, stents, and endoscopes (e.g., thinner stent delivery systems and ultra‐slim endoscopes that have an adequately sized working channel) is anticipated. All endoscopists must update themselves on the latest methods and equipment. Giving up quickly, especially in case of patients whose general condition is poor, should not be an option; instead, the aim should always be to decompress obstructive colorectal cancer with minimally invasive colonic stenting to prevent permanent stoma creation.

## CONFLICT OF INTEREST

Authors declare no conflict of interest for this article.

## FUNDING INFORMATION

None.
